# Editorial: Pharmacotherapy of energy metabolism in obesity

**DOI:** 10.3389/jpps.2025.14099

**Published:** 2025-03-10

**Authors:** John R. Ussher

**Affiliations:** ^1^ Faculty of Pharmacy and Pharmaceutical Sciences, University of Alberta, Edmonton, AB, Canada; ^2^ Alberta Diabetes Institute, University of Alberta, Edmonton, AB, Canada; ^3^ Cardiovascular Research Institute, University of Alberta, Edmonton, AB, Canada

**Keywords:** obesity, type 2 diabetes, cardiovascular disease, energy metabolism, pharmacotherapy

It is well established that body weight gain and increased adiposity during the progression of obesity increase one’s risk for metabolic dysfunction associated steatotic liver disease (MASLD), type 2 diabetes (T2D), and cardiovascular disease (CVD). Furthermore, a plethora of evidence supports that obesity leads to several perturbations in energy metabolism, which are widely thought to contribute to the pathology of these obesity-associated cardiometabolic pathologies [1]. Accordingly, researchers have invested significant effort towards understanding the molecular alterations that are responsible for these perturbations in energy metabolism, as they may represent novel drug discovery targets to treat MASLD, T2D, and CVD due to underlying obesity.

With the increasing recognition that the adipose tissue itself is a complex endocrine organ that secretes several cytokines and adipokines that can influence metabolic homeostasis, understanding how intermittent fasting mediated weight loss impacts whole-body energy metabolism is an exciting area for potential drug discovery Vo et al. Although not as prevalent as in rodents, there is also excitement on whether brown fat metabolism can be influenced in obese humans to promote weight loss Prapaharan et al. In addition, numerous metabolomics studies have demonstrated that increases in circulating branched-chain amino acids (leucine, isoleucine, valine) are positively associated with T2D and CVD [2]. Whether targeting BCAA metabolism can reverse obesity-related cardiometabolic disease has engendered significant interest in the field, though which organ (i.e., skeletal muscle, heart, adipose tissue, etc.) BCAA metabolism is most relevant to manipulate is still an area of ongoing debate Abdualkader et al.

More recently, perturbations in ketone metabolism have also been shown to contribute to obesity-associated cardiometabolic diseases [3, 4]. One of the most intriguing questions within this realm pertains to whether pharmacological augmentation of hepatic ketogenesis can alleviate MASLD Kwon et al., as increased ketogenesis would result in an elevation of fatty acid oxidation and subsequent decrease in hepatic lipid accumulation. This is a highly relevant area for current drug discovery, since MASLD is a major cause for abnormal liver function tests and is a pathology that currently has no approved therapies. Of interest, it is also gaining recognition that targeting energy metabolism within oxidative organs is not the only area for potential drug discovery, as the field of immunometabolism is one of the most rapidly expanding areas in obesity. This is due in part to increased adiposity often leading to a chronic low-grade inflammation that can contribute to T2D and CVD [5]. Whether perturbations in macrophage energy metabolism directly contribute to the chronic low-grade inflammation associated with obesity is unknown, and will be a key area for future interrogation that could lead to new exciting targets for drug development Wong et al.

Perturbations in energy metabolism are commonly observed in the myocardium during obesity/T2D, with several studies exploring whether targeting such perturbations can alleviate diabetic cardiomyopathy and/or heart failure [6]. Targets of interest include manipulating lipoprotein lipase activity to alleviate dyslipidemia and fatty acid supply to the myocardium Shang and Rodrigues, or manipulating the enzymatic machinery within cardiomyocytes to limit the accumulation of toxic lipids such as ceramides and diacylglycerols, thereby attenuating cardiac lipotoxicity Nakamura. One of the most robust metabolic perturbations in the myocardium during obesity/T2D is an impairment in glucose oxidation, with several studies illustrating that inhibition of the transcription factor, forkhead box O1, can restore glucose oxidation in the diabetic heart Shafaati and Gopal. There is also significant interest in understanding how acetylation of the enzymatic machinery controlling mitochondrial β-oxidation contributes to the elevations in myocardial fatty acid oxidation rates observed in obesity/T2D Ketema and Lopaschuk.

This special issue of the *Journal of Pharmacy and Pharmaceutical Sciences* features several topical review articles addressing these exciting areas of energy metabolism and how they have contributed to the potential development of new drugs for treating obesity-related cardiometabolic diseases. While targeting energy metabolism may prove fruitful towards alleviating MASLD, T2D, and/or CVD associated with obesity, there is also much excitement with the use of glucagon-like peptide-1 receptor (GLP-1R) agonists to directly treat obesity via decreasing appetite. Indeed, the significant weight loss resulting from GLP-1R agonist therapy has been shown to have salutary actions against cardiometabolic disease. As such, this special issue also addresses the expanding role of GLP-1R agonists to treat obesity, while contrasting the efficacy of these agents against that of bariatric surgery Morissette and Mulvihill. Furthermore, the prevalence of obesity is increasing in our adolescent population, and thus the potential pharmacotherapy of adolescent obesity will also be discussed Son. Taken together, an advanced understanding of the metabolic perturbations associated with obesity has the potential to provide several new drug targets for treating cardiometabolic diseases such as MASLD, T2D and CVD, while improving the quality of life for millions of individuals.
